# Insights into the Regulation of Rhizosphere Bacterial Communities by Application of Bio-organic Fertilizer in *Pseudostellaria heterophylla* Monoculture Regime

**DOI:** 10.3389/fmicb.2016.01788

**Published:** 2016-11-16

**Authors:** Linkun Wu, Jun Chen, Hongmiao Wu, Xianjin Qin, Juanying Wang, Yanhong Wu, Muhammad U. Khan, Sheng Lin, Zhigang Xiao, Xiaomian Luo, Zhongyi Zhang, Wenxiong Lin

**Affiliations:** ^1^College of Life Sciences, Fujian Agriculture and Forestry UniversityFuzhou, China; ^2^Key Laboratory of Crop Ecology and Molecular Physiology, Fujian Agriculture and Forestry UniversityFuzhou, China; ^3^College of Crop Science, Fujian Agriculture and Forestry UniversityFuzhou, China; ^4^Fujian Provincial Key Laboratory of Agroecological Processing and Safety Monitoring, Fujian Agriculture and Forestry UniversityFuzhou, China

**Keywords:** *Pseudostellaria heterophylla*, replant disease, bio-organic fertilizer, microbial community, deep pyrosequencing

## Abstract

The biomass and quality of *Pseudostellariae heterophylla* suffers a significant decline under monoculture. Since rhizosphere miobiome plays crucial roles in soil health, deep pyrosequencing combined with qPCR was applied to characterize the composition and structure of soil bacterial community under monoculture and different amendments. The results showed compared with the 1st-year planted (FP), 2nd-year monoculture of *P. heterophylla* (SP) led to a significant decline in yield and resulted in a significant increase in *Fusarium oxysporum* but a decline in *Burkholderia* spp. Bio-organic fertilizer (MT) formulated by combining antagonistic bacteria with organic matter could significantly promote the yield by regulating rhizosphere bacterial community. However, organic fertilizer (MO) without antagonistic bacteria could not suppress *Fusarium* wilt. Multivariate statistics analysis showed a distinct separation between the healthy samples (FP and MT) and the unhealthy samples (SP and MO), suggesting a strong relationship between soil microbial community and plant performance. Furthermore, we found the application of bio-organic fertilizer MT could significantly increase the bacterial community diversity and restructure microbial community with relatively fewer pathogenic *F. oxysporum* and more beneficial *Burkholderia* spp. In conclusion, the application of novel bio-organic fertilizer could effectively suppress *Fusarium* wilt by enriching the antagonistic bacteria and enhancing the bacterial diversity.

## Introduction

*Pseudostellaria heterophylla*, belonging to the family *Caryophyllaceae*, is highly valued in traditional Chinese medicine. It provides cures for ailments including anorexia, spleen deficiency, and palpitations because of its various active components including saponins, polysaccharides, and cyclopeptides (Zhao W.O. et al., 2015). It is mainly produced in Ningde City, Fujian Province, southeast China, which is known as a geo-authentic production zone with the most suitable soil and climate conditions for *P. heterophylla*. However, consecutive monoculture of this plant has led to a serious decline of biomass and quality of its underground tubers. Fields used for *P. heterophylla* cultivation can only be replanted once every 4 years (Wu H. et al., 2016; Wu L. et al., 2016). In addition, farmers generally apply a copious amount of pesticides and fertilizers to maintain production levels under this monoculture regime, but such application raises production costs, causes excessive pesticide residues, and leads to environmental pollution. In recent years, the market demand for *P. heterophylla* has forced farmers to plant the crop in fields outside of the geo-authentic production areas. However, *P. heterophylla* produced in these areas cannot be assured of quality because of the unsuitable environmental conditions (Wu et al., 2013). Therefore, it has become a priority to explore the mechanisms of consecutive monoculture problems, also known as replant disease, and strategies to effectively control these problems in *P. heterophylla*.

A growing body of evidence suggests that plant-microbe interactions play crucial roles in soil quality and crop health (Nunan et al., 2005; Lakshmanan et al., 2014; Macdonald and Singh, 2014). Xiong et al. (2015a) found that long-term consecutive monoculture of black pepper (*Piper nigrum* L.) led to a significant decline in soil bacterial abundance, especially in the *Pseudomonas* spp. Li et al. (2014) attributed the problems associated with consecutive monoculture in peanut to the changes in the structure of the soil microbial community induced by root exudates rather than to direct allelopathy. Wu L. et al. (2016) found that *P. heterophylla* monoculture can significantly increase the amount of *Fusarium oxysporum* in the rhizosphere, and root exudates could promote the growth of this soil-borne pathogen. Besides, previous studies demonstrated that a decrease in soil microbial diversity or the population size of antagonistic bacteria could result in the occurrence of soil-borne diseases (Latz et al., 2012; Shen et al., 2014). Mendes et al. (2011) used a PhyloChip-based metagenomic approach to compare the rhizospheric microbiomes of disease-suppressive and disease-conducive soils and indicated that the abundance of particular bacterial taxa including *Burkholderiaceae, Pseudomonadaceae*, and *Xanthomonadales* was more abundant in suppressive soil than in conducive soil and was more abundant in transplantation soil (conducive soil + 10% suppressive soil) than in the conducive soil. Therefore, increasing attention has been paid to the roles of soil microbial ecology in causing and controlling of replant disease (Mazzola and Manici, 2012; Köberl et al., 2013; Philippot et al., 2013; Cha et al., 2016). Unfortunately, however, few studies have been carried out to understand the relationship between the soil bacterial community and replant disease of *P. heterophylla*, as well as the methods to overcome replant disease of this plant.

Manipulation of the rhizosphere microflora to favor beneficial microorganisms was considered to be an effective approach in suppressing the soil-borne pathogens and recovering the microbial populations damaged by pathogens (Babalola, 2010; Qiu et al., 2012). Nadhrah et al. (2015) found that formulated bio-organic fertilizer containing *Burkholderia* GanoEB2 could effectively suppress basal stem rot in oil palm caused by the fungal pathogen *Ganoderma boninense*. Pal et al. (2001) found that plant growth promoting rhizobacteria (*Bacillus* spp. and *Pseudomonas* sp. EM85) were strongly antagonistic to *Fusarium moniliforme, Fusarium graminearum* and *Macrophomina phaseolina*, causal agents of maize root diseases, and *Bacillus* spp. MRF was also found to produce IAA, solubilized tri-calcium phosphate and fixed nitrogen from the atmosphere. Qiu et al. (2012) indicated that a bio-organic fertilizer which was a combination of manure compost with antagonistic microorganisms (*Bacillus* sp., *Paenibacillus* sp., etc.) was an effective approach to suppress *Fusarium* wilt of cucumber plants by regulating the microbial community of rhizosphere soil. Previous studies demonstrated that beneficial microorganisms could better colonize plant roots and protect plants from soil-borne diseases when they were applied to the soil with organic matter (OM) or manure (El-Hassan and Gowen, 2006; Yang et al., 2011; Yuan et al., 2014). The application of OM or manure can enhance the suppression of soil-borne diseases possibly by providing nutrients for the growth of beneficial microorganisms, by regulating the physicochemical property in the soil, or by inducing changes in the structure of the soil microbial community (Gorissen et al., 2004; Shen et al., 2014; Wen et al., 2015). Therefore, in this study, several beneficial bacteria (*Burkholderia* spp., *Bacillus* spp.) that have shown strong antagonistic activities against *F. oxysporum* were used as inocula to fortify organic fertilizer for the purpose of suppressing *P. heterophylla Fusarium* wilt (Supplementary Figure S1). In addition, our previous studies found that phenolic acids in the root exudates of medicinal plants mediated the increase of soil-borne pathogens (Wu H. et al., 2016; Wu L. et al., 2016). Some *Bacillus* spp. with a capacity of phenolic acid degradation (Supplementary Figure S2) were selected as the experimental strains for bio-organic fertilizer fermentation in order to degrade the phenolic acids released by *P. heterophylla*.

In the current study, we examined the shifts in bacterial communities under different soil amendments by using deep pyrosequencing combined with DNA barcoding. The objective of this investigation is to consider in detail the changes in diversity and composition of soil bacterial communities under *P. heterophylla* monoculture and explore the efficiency and underlying mechanisms of a novel bio-organic fertilizer in controlling replant disease of this plant.

## Materials and Methods

### Fertilizer Preparation and Application

The microbial fertilizer NO.2 used in this study was a mixture of the OM, the effective microorganisms and antagonistic bacteria (*Burkholderia* spp. including *Burkholderia* sp. 4, *Burkholderia* sp. 8; *Bacillus* spp. including *Bacillus pumilus, Bacillus cereus*, etc.), which were previously isolated and stored in our laboratory. In particular, *Burkholderia* sp. 4 and *Burkholderia* sp. 8 possess the *prnD* gene (KX885433, KX894563), responsible for the production of the antifungal compound pyrrolnitrin (PRN), and show very strong antagonistic activities against *F. oxysporum*, an agent known to cause wilt and rot disease of *P. heterophylla* (Supplementary Figure S1). *Bacillus pumilus* was found to promote plant growth (unpublished data). Besides, some *Bacillus* spp. including *Bacillus cereus, Bacillus subtilis* with a capacity of phenolic acids degradation (Supplementary Figure S2) were used as inocula to ferment OM for preparing the novel bio-organic fertilizer. The effective microorganisms and antagonistic bacteria were incubated separately in the liquid-state fermentation and then mixed together for solid-state fermentation. The OM consists of soybean meal and fish meal with 2:1 weight ratio. After solid-state fermentation using the OM as a substrate (30% moisture content) at 37°C for 48 h and then at room temperature for 4 weeks, the microbial fertilizer contained 1.0 × 10^9^ ∼ 5.0 × 10^9^ CFU g^-1^ of antagonistic bacteria. The fertilizer NO.1 was just the OM with the same fermentation process as microbial fertilizer NO.2 except the beneficial microbial inocula. When treating the field plots, the solid-state fermented fertilizer was completely mixed with soils, flooded with water and then mulched with black film for 1 month.

### Experiment Design and Soil Sampling

**FIGURE 1:**
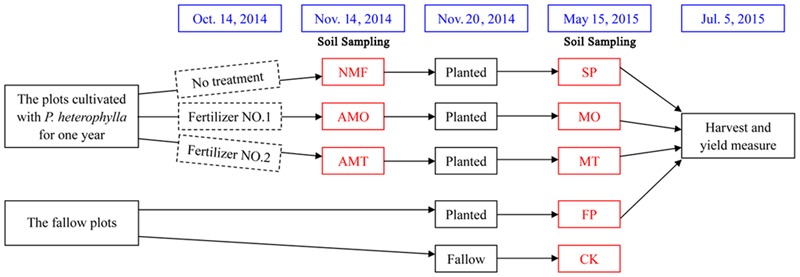
**The experimental design and soil sampling time.** AMO, AMT represent the 1-year cultivated plots that treated with equal amounts of microbial fertilizers NO.1, NO.2 for 1 month, respectively. NMF represents the 1-year cultivated plots without microbial fertilizer treatment and was sampled at the same time as AMO and AMT. CK, FP, and SP represent the control with no *Pseudostellaria heterophylla* cultivation, the 1st-year planted and 2nd-year monocultured plots, respectively. MO and MT represent the plots treated with microbial fertilizers NO.1 and NO.2 for 7 months, respectively.

The *P. heterophylla* ‘Zheshen 2,’ a cultivar widely planted in the geo-authentic production zones, was selected as the experimental material. The experiment was conducted at Xiapu County, Ningde City, Fujian Province, China (27° 08′ N, 119° 88′ E), with an annual mean precipitation of 1550 mm and annual mean temperature of 16°C. A field previously cultivated with rice (*Oryza sativa*) was used for experiments. The experimental design and sampling time are shown in **Figure 1**. More specifically, CK, FP and SP represent the control with no *P. heterophylla* cultivation, the 1st-year planted and 2nd-year monocultured plots, respectively. AMO, AMT represent the 1-year cultivated plots that treated with equal amounts of fertilizers NO.1, NO.2 for 1 month. NMF represent non-microbial fertilizer, namely the 1-year cultivated plots without microbial fertilizer treatment and was sampled at the same time as AMO and AMT. MO and MT represent the plots treated with microbial fertilizers NO.1 (Microbial fertilizer NO. ONE, MO) and NO.2 (Microbial fertilizer NO. Two, MT) for 7 months. Each treatment had three replicate plots and the study plots were completely randomized. To ensure the accuracy and repeatability, all treatments were organized within a single field site with the same climatic conditions and subjected to the same fertilization protocol and field management during the whole experimental period.

**FIGURE 2:**
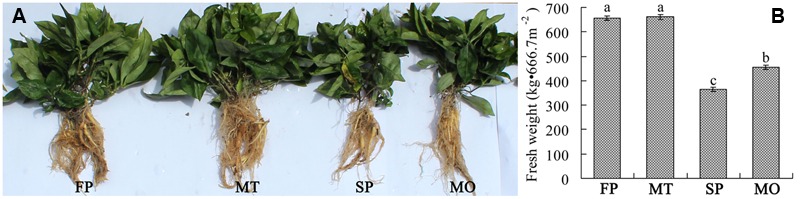
**Growth status (A)** and yield **(B)** of *P. heterophylla* under different treatments. FP and SP represent the 1st-year planted and 2nd-year monocultured plots, respectively. MO and MT represent the plots treated with microbial fertilizers NO.1 and NO.2 for 7 months. Data are mean ± standard deviation (one-way analysis of variance, *n* = 3). Different letters in B show significant differences determined by Tukey’s test (*P* ≤ 0.05).

After 1 month of microbial fertilizer application (November 14, 2014), soil physical and chemical properties (temperature, moisture, electrical conductivity, pH, and redox potential) were detected *in situ* by using HH2 Moisture Meter (Delta-T Devices, Ltd, England), pH meter (Spectrum Technologies, Inc., USA) and ORP Depolarization Automatic Analyzer (Nanjing Chuan-Di Instrument & Equipment Co., Ltd, China). And then the soils were sampled (NMF, AMO, and AMT) from five random locations for each treatment. At the expansion stages of tuberous roots (May 15, 2015), rhizosphere soils of *P. heterophylla* (FP, SP, MO, and MT) as well as control soils (fallow, CK) were sampled because of the pronounced difference in growth status between different treatments on this date (**Figure 2**). The rhizosphere soil tightly attached to tuberous roots of *P. heterophylla* was brushed off and collected. The collected soils were sieved through 2 mm mesh and immediately used for total soil DNA extraction. The rest of the soil samples were air-dried and used to determine soil nutrients (Wu et al., 2013). Briefly, available nitrogen (AN) was determined by alkaline hydrolysis method and total N (TN) was measured by Kjeldahl method. Available phosphorus (AP) was extracted using 0.5 mol/L NaHCO_3_ and then determined by Mo–Sb colorimetry method. Available potassium (AK) was extracted using CH_3_COONH_4_ and then measured by flame atomic absorption spectrometry. The total P and K (TP and TK) was calculated by first digesting the soil using the H_2_SO_4_–HClO_4_ and then measuring the level as described for AP and AK.

### DNA Extraction and Deep Pyrosequencing

For each soil sample, the extraction of total soil DNA was carried out in three replicates using a BioFast soil Genomic DNA Extraction kit (BioFlux, Hangzhou, China) following the manufacturer’s instructions. The DNA quality was monitored on 1% agarose gels. The DNA concentration was determined using a Nanodrop 2000C Spectrophotometer (Thermo Scientific, USA). Variable regions 3 to 4 (V3–V4) of bacterial 16S rRNA gene were amplified with the specific primers 341F (5′-CCTAYGGGRBGCASCAG-3′) and 806R (5′-GGACTACNNGGGT ATCTAAT-3′). All polymerase chain reaction (PCR) reactions were carried out using Phusion^®^ High-Fidelity PCR Master Mix (New England Biolabs).

The PCR products of each sample were pooled in equimolar concentrations and purified using a Qiagen Gel Extraction Kit (Qiagen, Germany). Sequencing libraries were generated with TruSeq^®^ DNA PCR-Free Sample Preparation Kit (Illumina, USA) following the manufacturer’s instructions, and then sequenced on an Illumina HiSeq2500 platform.

### Operational Taxonomic Unit (OTU)-Based Sequence Analysis

After pyrosequencing, raw sequences were assigned to the individual sample according to the unique barcode. The low-quality tags were excluded according to the QIIME (V1.7.0) quality-controlled process (Caporaso et al., 2010). Next, the chimera sequences were removed via the reference database (Gold database^1^) based on the comparison using the UCHIME algorithm (Edgar et al., 2011). Finally, the obtained effective tags were clustered into operational taxonomic units (OTUs) with a cutoff of 97% similarity. The singleton OTUs that contain only one sequence in all 12 samples were removed before further analysis. The remaining sequences were taxonomically classified using Ribosomal Database Project (RDP) classifier (Version 2.2) (Wang et al., 2007) via the GreenGene Database^2^.

### Statistical Analyses of Pyrosequencing Data

After data normalization, alpha and beta diversities were calculated to analyze the complexity of species diversity within a sample and differences between samples, respectively (Xiong et al., 2015b). An OTU-based analysis was carried out to calculate the following five indices (alpha diversity): observed-species (S_obs_), Chao1, ACE (Abundance-based Coverage Estimator), Shannon and Simpson diversity indices (Sun et al., 2014). Chao1 and ACE were calculated to estimate the richness of each sample (Chao, 1984; Shen et al., 2014). Shannon and Simpson indices were calculated to estimate the diversity within each individual sample (Lemos et al., 2011; Chen et al., 2012). A weighted UniFrac distance based on the abundance of lineages was calculated to analyze the differences in overall bacterial community composition and structure (beta diversity). Principal coordinate analysis (PCoA) and the unweighted pair-group method with arithmetic means (UPGMA) clustering was performed based on the weighted UniFrac distance.

One-way analysis of variance (ANOVA) followed by the Tukey’s test (*P* < 0.05, *n* = 3) was carried out for multiple comparisons. Spearman correlation coefficients between dominant bacterial taxa and yield of tuberous roots were calculated using SPSS v20.0 (SPSS, Inc., USA). Analysis of similarity (ANOSIM) was used to examine the statistical significance between samples. Similarity percentage (SIMPER) analysis was conducted to assess the relative contribution (%) of each taxon to the dissimilarity between samples. Both ANOSIM and SIMPER analyses were carried out in three replicates by using the PRIMER V5 software package (PRIMER-E, Ltd, UK) (Rees et al., 2004).

### Quantitative PCR for Genus *Burkholderia, F. oxysporum* and *prnD* Gene

Quantitative PCR (qPCR) was carried out to quantify the *Burkholderia, F. oxysporum and prnD gene* in different soil samples by using the taxon-specific primers (Supplementary Table S1). The reaction mixture (15 μl) for qPCR consists of 7.5 μl 2× SYBR green I SuperReal Premix (TIANGEN, Beijing, China), 0.5 μl of each primer (10 μM) and template DNA (20∼40 ng of total soil DNA or a serial dilution of plasmid DNA for standard curves). Three independent quantitative PCR assays were performed for each treatment.

## Results

### Effects of Consecutive Monoculture and Bio-organic Fertilizer Application on the Growth of *P. heterophylla*

The yields of *P. heterophylla* tuberous roots were detected under consecutive monoculture and bio-organic fertilizer application. Consecutive monoculture of *P. heterophylla* (SP) led to a serious decline in the above- and below-ground biomass, and an increase in pest and disease problems. When the 1-year cultivated plots were treated with the microbial fertilizer NO.2 (MT), the growth status of *P. heterophylla* greatly improved as compared with the 2nd-year monocultured plots (SP). However, the fertilizer NO.1 (MT) could not increase the yield of *P. heterophylla* tuberous roots. The fresh weights of tuberous roots in the 1st-year planted plots (FP), MT, SP, and MO were 656.3 kg/666.7 m^2^, 661.1 kg/666.7 m^2^, 364.3 kg/666.7 m^2^, and 400.7 kg/666.7 m^2^, respectively (**Figure 2**).

### Soil Physical and Chemical Properties of Different Treatments

**FIGURE 3:**
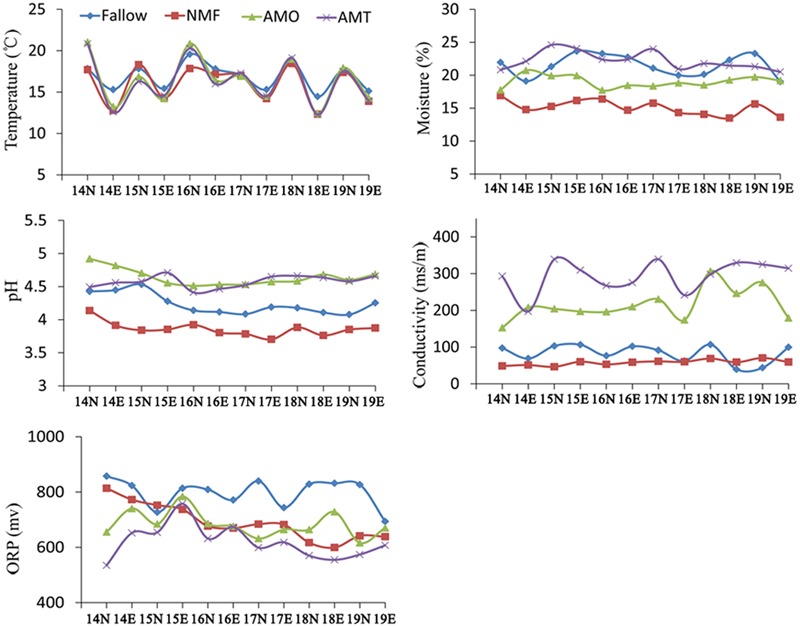
**Soil physical and chemical properties of different treatments.** Fallow represents the fallow plots with no *P. heterophylla* cultivation before. NMF represents the 1-year cultivated plots without microbial fertilizer treatment. AMO, AMT represent the 1-year cultivated plots that treated with microbial fertilizers NO.1, NO.2 for 1 month. The number in x-axis means the date from November 14 to 19, 2014. N and E followed by the date in x-axis represent the measure of soil physical-chemical properties performed at 1:00 PM and 6:00 PM, respectively. ORP represents soil redox potential. Data are means (*n* = 4).

One month after an application of microbial fertilizers, soil physical and chemical properties were detected *in situ*. The results showed that soils amended with microbial fertilizers for 1 month (AMO and AMT) had a higher level of moisture, pH, and electrical conductivity, especially for AMT. NMF had the lowest level of pH. Moreover, the fallow plots had the highest redox potential (ORP), while the soils amended with microbial fertilizer NO.2 had the lowest ORP value (**Figure 3**). The results suggest that application of microbial fertilizer produced by antagonistic bacteria could change the soil physical and chemical properties in a monoculture regime.

Furthermore, analysis of soil nutrients at the expansion stage of tuberous roots showed that the contents of available nitrogen (AN), available phosphorus (AP), and available potassium (AK) were significantly higher in SP than in FP and MT. Total nitrogen (TN) was significantly higher in FP and MT than in SP, while total phosphorus (TP) was significantly higher in SP than in FP and MT. Except for the AK and total potassium (TK), the rest of the soil nutrients were significantly higher in MO than in MT. TN, AN, and TK were significantly higher in FP than in MT while there was no significant difference in TP, AP, and AK between FP and MT (**Table 1**).

**Table 1:** Content of soil nutrients under five different treatments.

Treatments	TN (g/kg)	AN (mg /kg)	TP (g/kg)	AP (mg/kg)	TK (g/kg)	AK (mg/kg)
CK	1.74a	188.02cd	0.14d	26.10d	5.42b	99.81c
FP	1.65b	209.95b	0.19c	65.48c	5.79a	160.89b
SP	1.50d	260.74a	0.22b	73.02b	5.45b	272.43a
MO	1.77a	192.18c	0.28a	88.70a	5.25b	180.18b
MT	1.59c	183.67d	0.20c	65.61c	5.44b	181.26b

*CK, FP, and SP represent the control with no *P. heterophylla* cultivation, the 1st-year planted and 2nd-year monocultured plots, respectively. MO and MT represent the plots treated with microbial fertilizers NO.1 and NO.2 for 7 months, respectively. TN, AN, TP, AP, TK, and AK represent total nitrogen, available nitrogen, total phosphorus, available phosphorus, total potassium, available potassium, respectively. Different letters in columns show significant differences determined by Tukey’s test (*P* ≤ 0.05, *n* = 3).*

### OTU Cluster and Species Annotation

**FIGURE 4:**
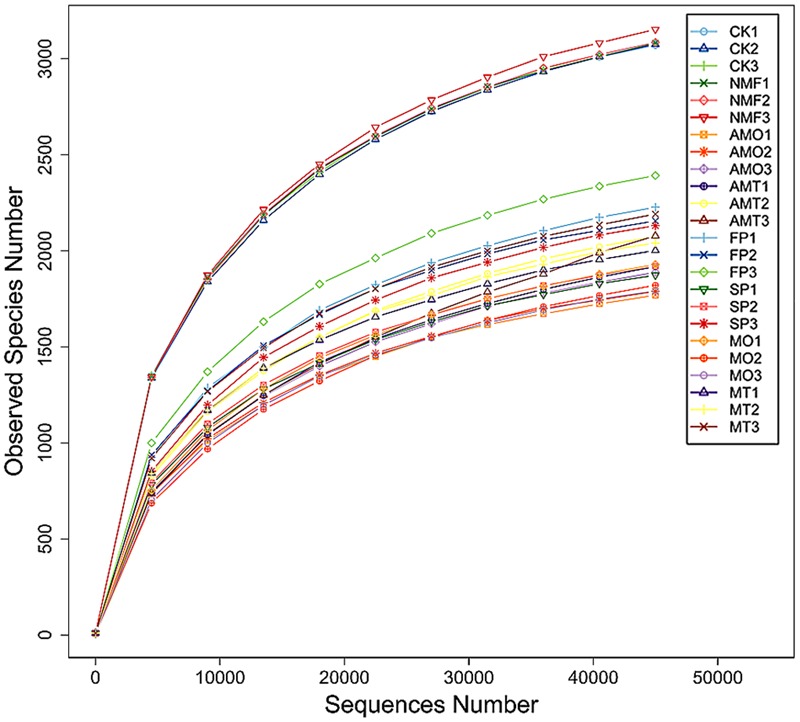
**Rarefaction curves of bacterial communities based on observed OTUs at 97% sequence similarity for individual samples.** CK, FP, and SP represent the control with no *P. heterophylla* cultivation, the 1st-year planted and 2nd-year monocultured plots, respectively. AMO, AMT represent the 1-year cultivated plots that treated with equal amounts of microbial fertilizers NO.1, NO.2 for 1 month, respectively. NMF represents the 1-year cultivated plots without microbial fertilizer treatment and was sampled at the same time as AMO and AMT. MO and MT represent the plots treated with microbial fertilizers NO.1 and NO.2 for 7 months, respectively. The numbers followed by the treatments represent the three replicates.

Deep 16S rRNA pyrosequencing was applied to assess the responses of the soil bacterial community to bio-organic fertilizer application in a *P. heterophylla* monoculture regime. Across all soil samples, a total of 1,233,132 effective tags with a species annotation were obtained, with an average of 54,502 effective tags per sample. In total, 74,900 singletons, accounting for 5.7% of total tags, were removed from the dataset before further analysis (Supplementary Figure S3). Rarefaction curves demonstrated that the number of observed OTUs tended to reach a plateau at 40,000 sequences (**Figure 4**). At a 97% sequence similarity cut-off, we obtained a sum of 62,464 OTUs across the 24 soil samples. The OTU numbers in CK, NMF, AMO, AMT, FP, SP, MO, and MT plots were 3,452, 3,482, 2,119, 2,269, 2,607, 2,281, 2,200, and 2,410, respectively (Supplementary Figure S3). On average, we were able to classify about 99.7% of effective sequences at the phylum level and 75.3% at the family level, but only 34.2% at the genus level (Supplementary Figure S4).

### Alpha Diversity Indices

Alpha diversity was calculated to analyze the complexity of species diversity within a sample. Among of them, the Chao1 estimator and the abundance-based coverage estimator (ACE) were calculated to estimate the richness of each sample. Both the Shannon and Simpson indices were calculated to estimate the diversity within each individual sample. In this study, the alpha diversity indices of soil bacterial community were calculated with a cutoff of 44,990 sequences. The bacterial community showed a significantly higher Shannon’s diversity index in FP than in SP (*P* < 0.05), and higher richness and Simpson’s indices (not significant, *P* > 0.05). Both the Shannon and Simpson diversity indices were significantly higher in MT than in MO (*P* < 0.05). The richness indices including the observed species, Chao1 and ACE indices, were higher in MT than in MO, but the difference was not significant (*P* > 0.05). No significant difference was observed in the Shannon and Simpson diversity indices between AMO and AMT (**Table 2**).

**Table 2:** Calculations of observed species, richness, and diversity in different soil samples.

Treatments	Observed species	Chao1	ACE	Shannon	Simpson
CK	3076.33a	3426.07a	3456.95a	9.65a	0.995a
FP	2258.33b	2547.92a	2627.19b	8.59b	0.989ab
SP	1974.67cd	2267.99a	2309.74b	7.78c	0.977b
NMF	3106.33a	3445.59a	3493.11a	9.66a	0.994a
AMO	1815.33d	2076.57a	2134.51b	7.79c	0.984ab
AMT	2024.00bcd	3151.08a	2745.65b	7.79c	0.985ab
MO	1847.00cd	2133.17a	2206.23b	7.07d	0.951c
MT	2078.00bc	2381.82a	2421.88b	8.37b	0.989ab

*CK, FP, and SP represent the control with no *P. heterophylla* cultivation, the 1st-year planted and 2nd-year monocultured plots, respectively. AMO, AMT represent the 1-year cultivated plots that treated with equal amounts of microbial fertilizers NO.1, NO.2 for 1 month, respectively. NMF represents the 1-year cultivated plots without microbial fertilizer treatment and was sampled at the same time as AMO and AMT. MO and MT represent the plots treated with microbial fertilizers NO.1 and NO.2 for 7 months, respectively. Different letters in columns show significant differences determined by Tukey’s test (*P* ≤ 0.05, *n* = 3).*

### Beta Diversity Indices

**FIGURE 5:**
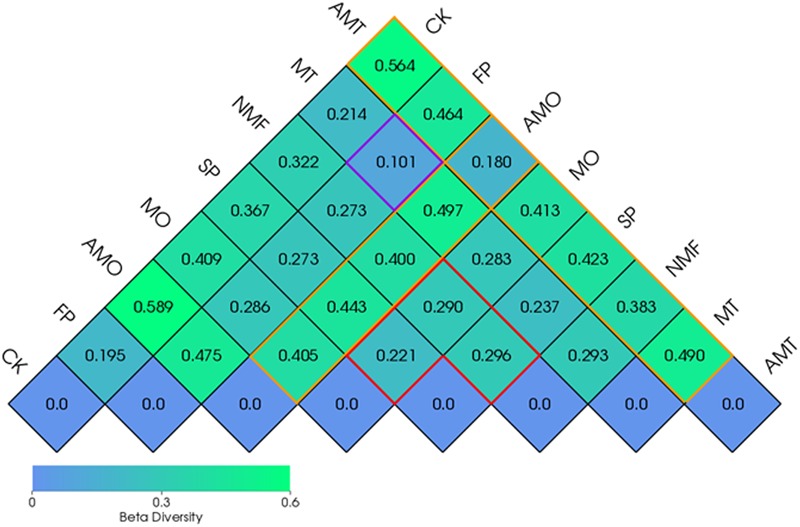
**Weighted Unifrac distances between different soil samples.** CK, FP, and SP represent the control with no *P. heterophylla* cultivation, the 1st-year planted and 2nd-year monocultured plots, respectively. AMO, AMT represent the 1-year cultivated plots that treated with equal amounts of microbial fertilizers NO.1, NO.2 for 1 month, respectively. NMF represents the 1-year cultivated plots without microbial fertilizer treatment and was sampled at the same time as AMO and AMT. MO and MT represent the plots treated with microbial fertilizers NO.1 and NO.2 for 7 months, respectively. Each treatment has three replicates.

Beta diversity was calculated to analyze the differences in overall bacterial community composition and structure between samples. In this study, the weighted Unifrac distance between FP and MT was a minimum of 0.101 (the purple outline), suggesting a high similarity of the soil bacterial community between these two healthy samples. However, a relatively great dissimilarity was observed between AMO and other treatments and between AMT and other treatments (the orange outline). The similarity among SP, NMF, and MO was high, as indicated by the low weighted Unifrac distances among each other (the red outline) (**Figure 5**).

### PCoA and UPGMA Clustering

**FIGURE 6:**
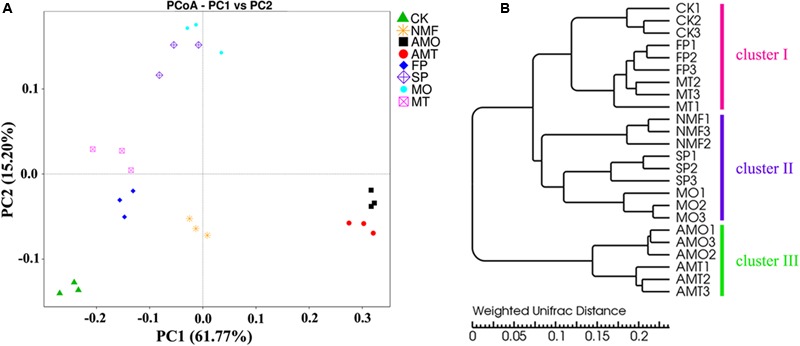
**Principal coordinate analysis (PCoA) (A)** and hierarchical clustering **(B)** of bacterial communities based on weighted Unifrac algorithm for different soil samples. CK, FP, and SP represent the control with no *P. heterophylla* cultivation, the 1st-year planted and 2nd-year monocultured plots, respectively. AMO, AMT represent the 1-year cultivated plots that treated with equal amounts of microbial fertilizers NO.1, NO.2 for 1 month, respectively. NMF represents the 1-year cultivated plots without microbial fertilizer treatment and was sampled at the same time as AMO and AMT. MO and MT represent the plots treated with microbial fertilizers NO.1 and NO.2 for 7 months, respectively. The numbers followed by the treatments represent the three replicates.

To compare bacterial community structures across all samples, PCoA and UPGMA clustering were performed based on the weighted UniFrac distance. The PCoA analysis based on the weighted Unifrac distance revealed distinct differences in soil bacterial community structure between different treatments. The first two components (PC1 and PC2) of PCoA explained 61.77 and 15.20% of the total bacterial community variations, respectively. The bacterial communities of the MO-treated (MO) soil and the 2nd-year monoculture soil (SP) (both unhealthy samples) formed a separate group. However, the bacterial communities of the MT-treated soil (MT) and the 1st-year cultivated soil (FP) (both healthy samples) formed a separate group, which were distinctly different from those in the unhealthy samples (**Figure 6A**).

Furthermore, UPGMA clustering showed similar bacterial community structure for the same treatment in triplicate and obvious differences between different treatments, as observed in the three highly supported clusters (clusters I, II, and III). Bacterial community structure from FP, MT, and CK were clustered together (cluster I) and were separated from the soil samples from SP, MO, and NMF, which were grouped together (cluster II). The bacterial community structure from soil samples that were amended with microbial fertilizers NO.1 and NO.2 for 1 month clustered together (cluster III), which was distinguished from cluster I and cluster II (**Figure 6B**). The results from PCoA and UPGMA cluster analyses indicated that microbial fertilizer NO.2 could repair the imbalance in the bacterial community in replanted soils, but the microbial fertilizer NO.1 could not create a healthy bacterial community structure, although, it altered the bacterial community after 1 month of treatment.

### Venn Diagram Analysis

**FIGURE 7:**
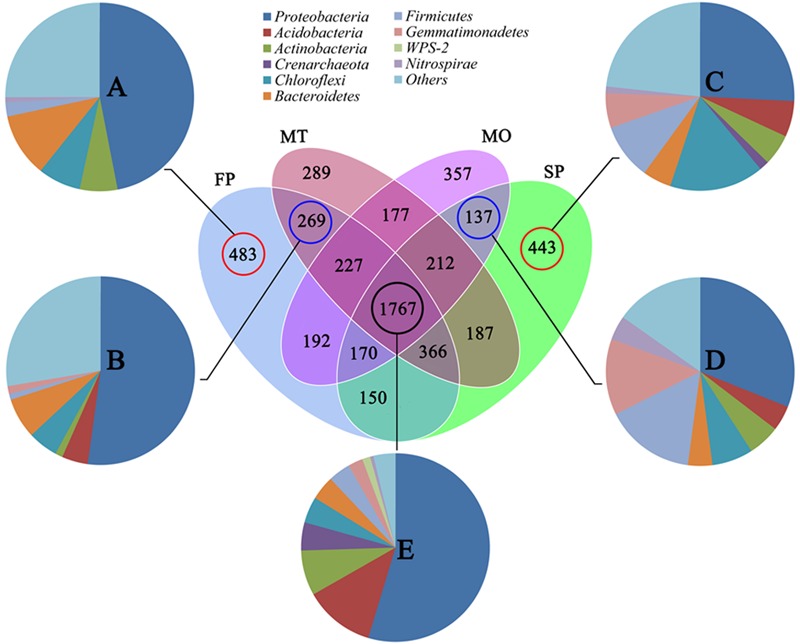
**Venn diagram of exclusive and shared species-level taxa among the 1st-year planted (FP), 2nd-year monocultured (SP), microbial fertilizers NO.1 (MO) and NO.2 (MT)-treated soils. (A)** Pie chart of exclusive OTUs (483) in FP; **(B)** Pie chart of exclusive OTUs (443) in SP; **(C)** Pie chart of exclusive OTUs (269) shared between FP and MT based on the average abundance of each phylum. **(D)** Pie chart of exclusive OTUs (137) shared between SP and MO based on the average abundance of each phylum. **(E)** Pie chart of shared OTUs (1767) among FP, SP, MO, and MT based on the average abundance of each phylum. Each treatment has three replicates.

Venn diagram analysis was carried out to detect the exclusive and shared OTUs between the healthy (FP and MT) and unhealthy samples (SP and MO). The results showed that the number of OTUs exclusively found in FP was 483 (13.3%). The number of OTUs exclusively found in SP was 443 (12.9%). The number of OTUs only shared in FP and MT was 269 (6.0%), and that only shared in SP and MO was 137 (3.1%). Moreover, high similarity was observed between the pie charts A and B and between the pie charts C and D. The percentage of OTUs shared in FP, SP, MO, and MT was 32.6% (1,767 species), and these were mainly assigned to the class *Proteobacteria* (52.1%), *Acidobacteria* (11.5%), and *Actinobacteria* (7.4%) (**Figure 7**). The abundance of these 1,767 OTUs shared in the healthy and unhealthy samples accounted for up to 93.9, 96.4, 95.1, and 96.1% of the total detected taxa in FP, MT, SP, and MO plots, respectively.

### Shifts in Soil Bacterial Community Structure under Consecutive Monoculture

The taxonomy of OTUs in each sample was assigned using the Ribosomal Database Project (RDP) classifier via the GreenGene Database. The results showed that the bacterial OTUs were comprised mainly of 10 phyla, *Proteobacteria, Acidobacteria, Bacteroidetes, Firmicutes, Actinobacteria, Chloroflexi, Crenarchaeota, Gemmatimonadetes, Nitrospirae*, and *Verrucomicrobia*. *Proteobacteria* was the dominant microbial taxa, and accounted for approximately 50% of the total population in each sample. Compared with NMF, those bacteria belonging to *Firmicutes* and *Bacteroidetes* significantly increased after bio-organic fertilizer application (MO and MT), while *Acidobacteria, Chloroflexi*, and *Gemmatimonadetes* significantly decreased. When the treated soils were planted with *P. heterophylla* under aerobic conditions, those bacteria belonging to *Acidobacteria* and *Chloroflexi* significantly increased, but *Bacteroidetes* and *Firmicutes* significantly decreased (Supplementary Figure S5).

Analysis of similarity of the pyrosequencing data consisting of the relative abundance of genera (logarithmic transformation and standardization) showed that the bacterial communities differed significantly between eight different treatments (ANOSIM Global *R* = 0.977, *P* = 0.001). ANOSIM analysis based on the presence/absence of genera also showed a significant difference among eight treatments in bacterial communities (ANOSIM Global *R* = 0.901, *P* = 0.001). Furthermore, similarity percentage analysis (SIMPER) analysis using the relative abundance of genera (logarithmic transformation and standardization) showed that the pairwise dissimilarity in bacterial communities between MO and MT was 26.57%, and was 28.34% between FP and SP. The top shared genera that contributed approximately 60% to the observed differences between MO and MT and between FP and SP included *Hylemonella, Candidatus koribacter, Dokdonella, Dyella, Rhodoplanes, Burkholderia, Kaistobacter*, and *Clostridium* (**Table 3**). Moreover, the relative abundances of *Burkholderia, Kaistobacter*, and *Clostridium* were significantly (*P* < 0.01) positively correlated with the yield of *P. heterophylla* tuberous roots (**Table 3**). Compared with the 2nd-year monocultured (SP) and microbial fertilizer NO.1 (MO) treatments, the relative abundance of genus *Burkholderia* in the microbial fertilizer NO.2 (MT) treatment increased by 453.7 and 116.0%, respectively.

**Table 3:** Top shared genera with 60% cumulative contribution to the dissimilarity between MO and MT, and between FP and SP.

Genus	Family	Phylum	Contribution (%)		Relative abundance (%)^§^				Spearman ρ^#^
			MO and MT	FP and SP	FP	SP	MO	MT	
*Hylemonella*	*Comamonadaceae*	β-*Proteobacteria*	8.64	4.69	0.00c	0.69b	1.61a	0.07c	-0.600
*Candidatus koribacter*	*Koribacteraceae*	*Acidobacteria*	4.70	2.98	1.50a	0.79bc	0.37c	1.22ab	0.600
*Dokdonella*	*Xanthomonadaceae*	γ-*Proteobacteria*	3.12	4.22	0.18c	0.88a	0.37b	0.89a	0.200
*Dyella*	*Xanthomonadaceae*	γ-*Proteobacteria*	3.04	3.40	0.75a	0.20c	0.18c	0.61b	0.600
*Rhodoplanes*	*Hyphomicrobiaceae*	α-*Proteobacteria*	2.57	2.85	1.79a	1.03b	1.03b	1.64a	0.600
*Burkholderia*	*Burkholderiaceae*	β-*Proteobacteria*	2.29	1.44	0.31b	0.12c	0.30b	0.64a	1.000^∗∗^
*Kaistobacter*	*Sphingomonadaceae*	α-*Proteobacteria*	1.82	4.83	2.51a	1.05b	2.19a	2.73a	1.000^∗∗^
*Clostridium*	*Clostridium*	*Firmicutes*	1.44	1.56	0.29b	0.08c	0.21b	0.39a	1.000^∗∗^

*Similarity percentage (SIMPER) analysis was conducted to assess the relative contribution (%) of each taxon to the dissimilarity between samples (MO and MT, FP and SP) based on the relative abundance of each genus (logarithmic transformation, standardization). FP and SP represent the 1st-year planted and 2nd-year monocultured plots, respectively. MO and MT represent the plots treated with microbial fertilizer NO.1 and NO.2 for 7 months. ^§^ Different letters within a line show significant differences determined by Tukey’s test (*P* ≤ 0.05, *n* = 3). ^#^Spearman correlation coefficients between the abundance of bacterial taxa and the yield of *P. heterophylla* tuberous roots. ^∗∗^ mean *P* < 0.01, respectively.*

### Quantification of *F. oxysporum*, Genus *Burkholderia*, and the *prnD* Gene

**FIGURE 8:**
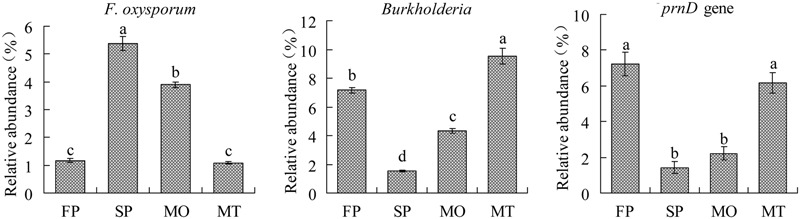
**Quantification of *Fusarium oxysporum, genus Burkholderia, prnD gene* in four different soil samples.** FP and SP represent the 1st-year planted and 2nd-year monocultured plots, respectively. MO and MT represent the plots treated with microbial fertilizers NO.1 and NO.2 for 7 months, respectively. Data are mean ± standard deviation (one-way analysis of variance, *n* = 3). Different letters show significant differences determined by Tukey’s test (*P* ≤ 0.05).

The amounts of genus *Burkholderia*, potential biocontrol agents, and *F. oxysporum*, the main agent causing rot disease of this plant were detected by quantitative PCR. The results showed that the relative abundance of *F. oxysporum* was significantly greater in the SP and MO (unhealthy soils) than in the FP and MT (healthy soils) while the abundance of *Burkholderia* was significantly lower in the SP and MO (unhealthy soils) than in the FP and MT (healthy soils) (**Figure 8**). The results were consistent with the deep pyrosequencing analysis (**Table 3**). However, application of microbial fertilizer NO.2 produced by antagonistic bacteria increased the amounts of beneficial *Burkholderia* spp. in the rhizosphere. Besides, quantitative PCR of *prnD*, a gene responsible for the production of the antibiotic pyrrolnitrin, showed that the population harboring *prnD* gene was significantly greater in the healthy soils (FP and MT) than in the unhealthy soils (SP and MO) (**Figure 8**).

## Discussion

The consecutive monoculture problem, also known as replant disease, commonly occurs in the cultivation of a range of crops in intensive agriculture, especially in the production of medicinal plants. Approximately 70% of medicinal plant species using tuberous roots have various degrees of consecutive monoculture problems (Zhang and Lin, 2009), which substantially limits the development of traditional Chinese medicines. Our field experiment showed that consecutive monoculture of *P. heterophylla* resulted in a significant decline in the yield of tuberous roots (**Figure 2**). Many factors have been thought to be responsible for the consecutive monoculture problem of *P. heterop*hylla, including soil physical and chemical properties, accumulation of allelochemicals released by roots, and shifts in the soil microbial community (Wu L. et al., 2016). In this study, we found that the content of most of the soil nutrients were significantly higher in the unhealthy samples (SP and MO) than in the healthy samples (NP and MT) (**Table 1**). Moreover, our study showed the utilization of OM without antagonistic bacteria (MO) in monocultured plots could improve soil nutrients but not effectively eliminate replant disease of this plant. However, the application of microbial fertilizers could significantly increase the pH value and soil electrical conductivity. The monocultured plots without microbial fertilizer treatment (NMF) were found to have the lowest pH (**Figure 3**). Previous studies have shown that consecutive monoculture of many plants could lead to a significant decrease in soil pH (Meriles et al., 2009; Wu et al., 2015). Many pathogenic fungi including *Fusarium* spp. and *Pythium* spp. are adapted to a slightly low pH (Husson, 2013). Both soil pH and electrical conductivity were recognized as important factors affecting microbial processes and ecology in soil (Gelsomino and Cacco, 2006; Hogberg et al., 2007). Additionally, application of microbial fertilizer NO.2 could significantly increase the soil electrical conductivity but decrease the soil ORP (**Figure 3**). OM was reported to play a dominant role in affecting soil ORP, leading to a lowering of soil ORP when soil OM increased (Husson, 2013). Many researchers have indicated that the development of several plant pathogens (*Fusarium* spp. and *Rhizoctonia solani*) were closely related to a high soil ORP (Takehara et al., 2004; Husson, 2013). Some agricultural practices used to reduce soil ORP including the application of decomposable organic material and flooding or covering the soil with plastic could effectively control soil-borne pathogens (Blok et al., 2000; Mowlick et al., 2012; Wen et al., 2015). Wen et al. (2015) found that biological soil disinfestations (BSD) conducted by incorporating organic amendments into the soil under flooding conditions could rapidly decrease the soil ORP, which would contribute to pathogen inactivation. The results mentioned above suggested that the soil physical and chemical properties may indirectly influence *P. heterophylla* performance by modulating the soil microbial community structure.

Since researchers indicated that allelochemicals released by roots were not sufficient to directly inhibit neighboring plants or the host plant, more attention has been paid to the below-ground microbial community structure and function diversity mediated by rhizodeposition (Berendsen et al., 2012; Chaparro et al., 2014; Haney and Ausubel, 2015; Xiong et al., 2016). In this study, deep pyrosequencing combined with the qPCR technique was used to assess the changes in the microbial community under monoculture and different amendments. The results demonstrated that consecutive monoculture of *P. heterophylla* led to a significant variation in the bacterial community structure in the rhizosphere, but the OM fermented by antagonistic bacteria (MT) could recover the bacterial community composition (**Figure 5**). It was found that the application of bio-organic fertilizer could significantly increase those bacteria belonging to *Firmicutes* and *Bacteroidetes* but significantly decrease *Acidobacteria* (Supplementary Figure S5), which was in accordance with previous reports (Mowlick et al., 2012; Huang et al., 2015). Furthermore, both PCoA analysis and UPGMA clustering showed a distinct separation between the healthy samples (FP and MT) and the unhealthy samples (SP and MO), suggesting the strong relationship between soil microbial community and plant performance (**Figure 6**). In addition, our findings showed that the healthy samples (FP and MT) had significantly higher bacterial community diversity than the unhealthy samples (SP and MO) (**Table 2**). A high microbial diversity was considered to be responsible for the development and maintenance of disease suppressive soils (Van Bruggen and Semenov, 2000; Qiu et al., 2012; Yuan et al., 2014). Many researchers showed that organic amendments could induce general suppression in the soil by increasing soil microbial activity, enhancing competition for nutrients and ecological niches available for soil-borne pathogens (Kotsou et al., 2004; Shen et al., 2014).

Moreover, we found that consecutive monoculture of *P. heterophylla* led to a significant increase in the relative abundance of *F. oxysporum* but a decrease in the potential beneficial bacteria including *Burkholderia* and *Clostridium* (Mowlick et al., 2012; Ho et al., 2015; Wen et al., 2016). Our previous studies by culture-dependent and culture-independent analysis demonstrated that *F. oxysporum* and other *Fusarium* spp. are the main causative agents of wilt and rot disease of this plant (Zhao Y. et al., 2015; Wu L. et al., 2016). Our previous studies have demonstrated that the phenolic acids identified in the root exudates of *P. heterophylla* could significantly inhibit the growth of beneficial *Burkholderia* sp. but promote the growth of soil-borne *F. oxysporum* (Lin et al., 2015; Wu L. et al., 2016). However, bio-organic fertilizer, in particular, MT containing antagonistic bacteria could rebalance the beneficial and detrimental microbial residents in soil, leading to relatively more beneficial microorganisms and relatively fewer pathogenic microorganisms (**Figure 8**; **Table 3**). Compared with the 2nd-year monocultured (SP) and microbial fertilizer NO.1 (MO) treatments, the relative abundance of genus *Burkholderia* in the microbial fertilizer NO.2 (MT) treatment increased by 453.7 and 116.0%, respectively. The increase might be mainly due to the organic amendments and the addition of *Bacillus* spp. with a capacity of degradation of phenolic acids into bio-organic fertilizer. The application of OM can provide nutrients for the growth of beneficial microorganisms and regulate the physicochemical property in the soil, which may be favorable for the growth of beneficial microorganism and contribute to pathogen inactivation (Husson, 2013; Shen et al., 2014; Wen et al., 2015). In this study, we also found that the application of OM could significantly increase soil pH but decrease soil redox potential (ORP) (**Figure 3**). However, it should be noted that we were only able to classify approximately 4.9% of the effective sequences at the species level based on the pyrosequencing data of hypervariable regions 3–4 (V3–V4, 420 bp) of the bacterial 16S rRNA gene (Supplementary Figure S4). The *Burkholderia* spp. added in the bio-fertilizer in this study could not been identified at the species level. Therefore, more studies on species-level classification and analysis are needed.

Biological control is considered to be the most promising technique for plant disease prevention by favoring beneficial microorganisms that are directly antagonistic to root pathogens (Qiu et al., 2012; Shen et al., 2014). A growing number of *Burkholderia* species have been reported to be beneficial for plants, including biological control of fungal diseases, promotion of plant growth and induction of systemic resistance (Compant et al., 2008; Ho et al., 2015). The *Burkholderia* strains used for solid fermentation in this study proved to have a strong antagonistic activity against *F. oxysporum* (Supplementary Figure S1), the main agent causing replant disease of *P. heterophylla* (Wu L. et al., 2016). Therefore, the mechanism underlying the reduction of replant disease by the application of *Burkholderia-*fortified organic fertilizer might be attributed not only to general suppression but also to specific suppression referring to an increase in the specific microorganisms with antagonistic activity against the specific pathogen (Kotsou et al., 2004). Pyrrolnitrin (PRN), a chlorinated phenylpyrrole antibiotic catalyzed by the *prnD* gene product, is produced by many strains of *Burkholderia* spp. (de Souza and Raaijmakers, 2003). In this study, quantitative PCR analysis of the *prnD* gene showed that consecutive monoculture of *P. heterophylla* led to a significant decline in the population harboring *prnD* gene. In contrast, the application of bio-organic fertilizer could significantly increase the amount of *prnD* gene in the monocultured soils (**Figure 8**). Latz et al. (2012) found that soil suppressiveness against pathogens by fostering beneficial bacterial communities was at maximum when the abundance of PRN and 2,4-diacetylphloroglucinol (DAPG) producer was high in soil.

## Conclusion

Consecutive monoculture of *P. heterophylla* can alter the microbial community in the rhizosphere, including enrichment of host-specific pathogens at the expense of beneficial microorganisms. However, application of bio-organic fertilizer could restructure the microbial community in the rhizosphere and effectively suppress *Fusarium* wilt by enriching the antagonistic bacteria and enhancing the bacterial diversity. Our results give a promising strategy for replant disease control by manipulating the rhizosphere microbiome to keep soil healthy and enhance *P. heterophylla* production and sustainability.

## Author Contributions

WL and LW conceived the study; LW, WL, JC, and MK wrote the paper. LW, JC, HW, XQ, JW, XL, and ZX performed experiments; LW, JC, and SL performed the statistical analyses; ZZ, YW, and JC are involved in field management and soil sampling. All authors discussed the results and commented on the manuscript.

## Conflict of Interest Statement

The authors declare that the research was conducted in the absence of any commercial or financial relationships that could be construed as a potential conflict of interest.

## Footnotes

1 http://drive5.com/uchime/uchime_download.html

2 http://greengenes.lbl.gov/cgi-bin/nph-index.cgi
