# Supplementary material for: Insights into the Regulation of Rhizosphere Bacterial Communities by Application of Bio-organic Fertilizer in Pseudostellaria heterophylla Monoculture Regime
Source: Front Microbiol. 2016 Nov 16;7:1788. doi: 10.3389/fmicb.2016.01788 (PMC5110535; doi:10.3389/fmicb.2016.01788)
Supplement: Supplementary file 1 [file Data_Sheet_1.DOC]

**Insights into the Regulation of Rhizosphere Bacterial Communities** **by Application of Bio-organic Fertilizer in *Pseudostellaria heterophylla* Monoculture Regime**

*Linkun Wu^1,2#^, Jun Chen^1,2#^, Hongmiao Wu^1,2^, Xianjin Qin^1,2^,* *Juanying Wang^1,2^, Yanhong Wu^1,2^, Muhammad Umar Khan^1,2^, Sheng Lin^1,2^, Xiaomian Luo^3,4^, Zhongyi Zhang^3,4^ and Wenxiong Lin**^1,3^**^*^*

*^1^ College of Life Sciences, Fujian Agriculture and Forestry University, Fuzhou, Fujian, China.*

*^2^ Key Laboratory of Crop Ecology and Molecular Physiology, Fujian Agriculture and Forestry University, Fuzhou, Fujian, China.*

*^3^ Fujian Provincial Key Laboratory of Agroecological Processing and Safety Monitoring, Fujian Agriculture and Forestry University, Fuzhou, Fujian, China.*

*^4^ College of Crop Science, Fujian Agriculture and Forestry University, Fuzhou, Fujian, China.*

*Correspondence:

Wenxiong Lin

[lwx@fafu.edu.cn](mailto:lwx@fafu.edu.cn)

^#^ These authors contributed equally to this work.

**SUPPLEMENTARY MATERIAL**

**Supplementary FIGURE S1 | Assessment of antagonistic activity of *Burkholderia* spp. (A, B and C) and *prnD* gene amplification (D).** FON represents *F. oxysporum*. *prnD* represents the gene responsible for the production of the antifungal compound pyrrolnitrin (PRN). The 7 numbers in D represent the DNA marker (1), *prnD* gene amplified from *Burkholderia* sp. 4, *Burkholderia* sp. 8, *Bacillus cereus*, *Bacillus pumilus*, *Pseudomonas* sp. 313, *Pseudomonas* sp. 361 (2～7), respectively.

**Supplementary FIGURE S2 | The consumption of phenolic acids by *Bacillus cereus* and *Bacillus subtilis*.**

**Supplementary FIGURE S3 | Statistics of OTU cluster and species annotation for individual samples.** Total Tags (red columns): represent the total number of effective tags; Unique Tags (orange columns): represent the total numbers of singletons, which were removed from the dataset before further analysis; Taxon Tags (blue columns): represent the total numbers of tags subjected to OTU cluster and with species annotation; Unclassified Tags (green columns): represent the total numbers of tags without species annotation; OTUs (purple columns): represent the OTUs numbers for each sample. CK, FP and SP represent the control with no *P. heterophylla* cultivation, the newly planted and two-year monocultured plots, respectively. AMO, AMT represent the one-year cultivated plots that treated with equal amounts of microbial fertilizers NO.1, NO.2 for one month, respectively. NMF represents the one-year cultivated plots without microbial fertilizer treatment and was sampled at the same time as AMO and AMT. MO and MT represent the plots treated with microbial fertilizers NO.1 and NO.2 for seven months, respectively.

**Supplementary FIGURE S4 | Sequence numbers for each taxonomic level in 24 soil samples.** CK, FP and SP represent the control with no *P. heterophylla* cultivation, the newly planted and two-year monocultured plots, respectively. AMO, AMT represent the one-year cultivated plots that treated with equal amounts of microbial fertilizers NO.1, NO.2 for one month, respectively. NMF represents the one-year cultivated plots without microbial fertilizer treatment and was sampled at the same time as AMO and AMT. MO and MT represent the plots treated with microbial fertilizers NO.1 and NO.2 for seven months, respectively.

**Supplementary FIGURE S5 | Relative abundances of the top 10 baterial phyla in eight different soil samples.** CK, FP and SP represent the control with no *P. heterophylla* cultivation, the newly planted and two-year monocultured plots, respectively. AMO, AMT represent the one-year cultivated plots that treated with equal amounts of microbial fertilizers NO.1, NO.2 for one month, respectively. NMF represents the one-year cultivated plots without microbial fertilizer treatment and was sampled at the same time as AMO and AMT. MO and MT represent the plots treated with microbial fertilizers NO.1 and NO.2 for seven months, respectively.

**Supplementary TABLE S1 | Taxon-specific primer sets and their annealing temperatures for quantitative PCR.**


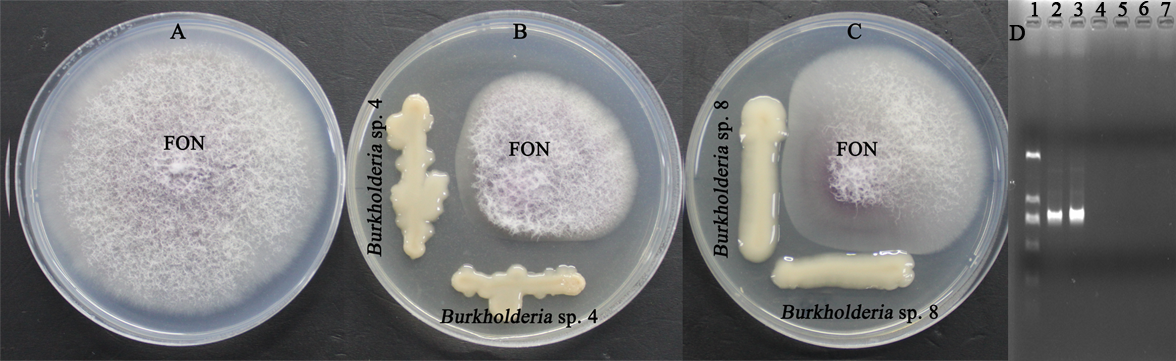


**Supplementary FIGURE S1 | Assessment of antagonistic activity of *Burkholderia* spp. (A, B and C) and *prnD* gene amplification (D).** FON represents *F. oxysporum*. *prnD* represents the gene responsible for the production of the antifungal compound pyrrolnitrin (PRN). The 7 numbers in D represent the DNA marker (1), *prnD* gene amplified from *Burkholderia* sp. 4, *Burkholderia* sp. 8, *Bacillus cereus*, *Bacillus pumilus*, *Pseudomonas* sp. 313, *Pseudomonas* sp. 361 (2～7), respectively.


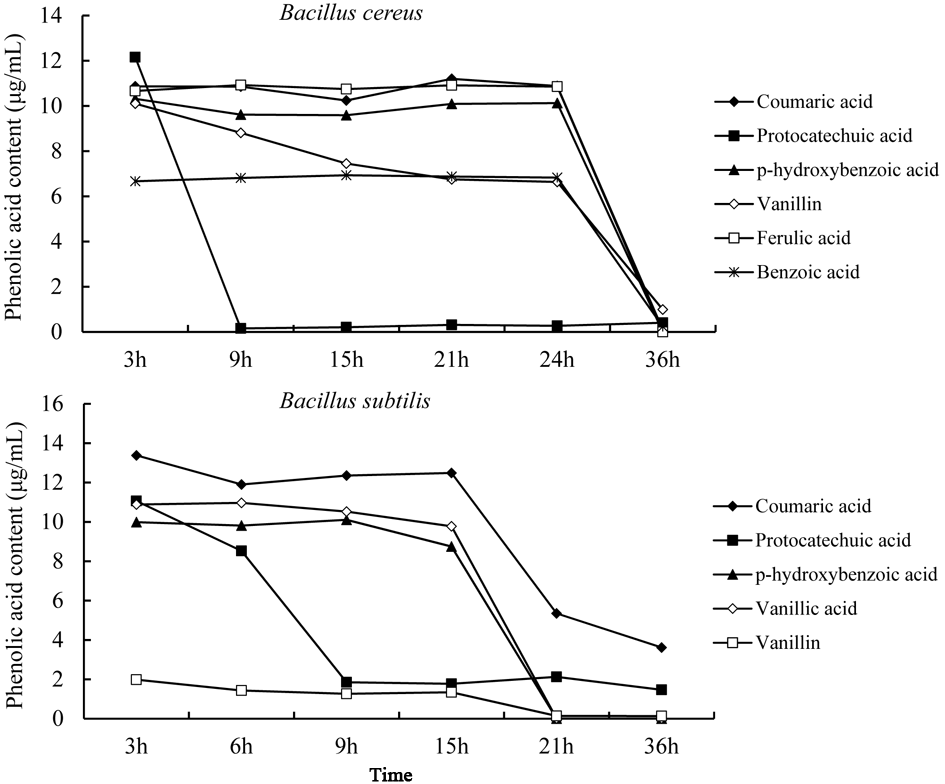


**Supplementary FIGURE S2 | The consumption of phenolic acids by *Bacillus cereus* and *Bacillus subtilis*.**


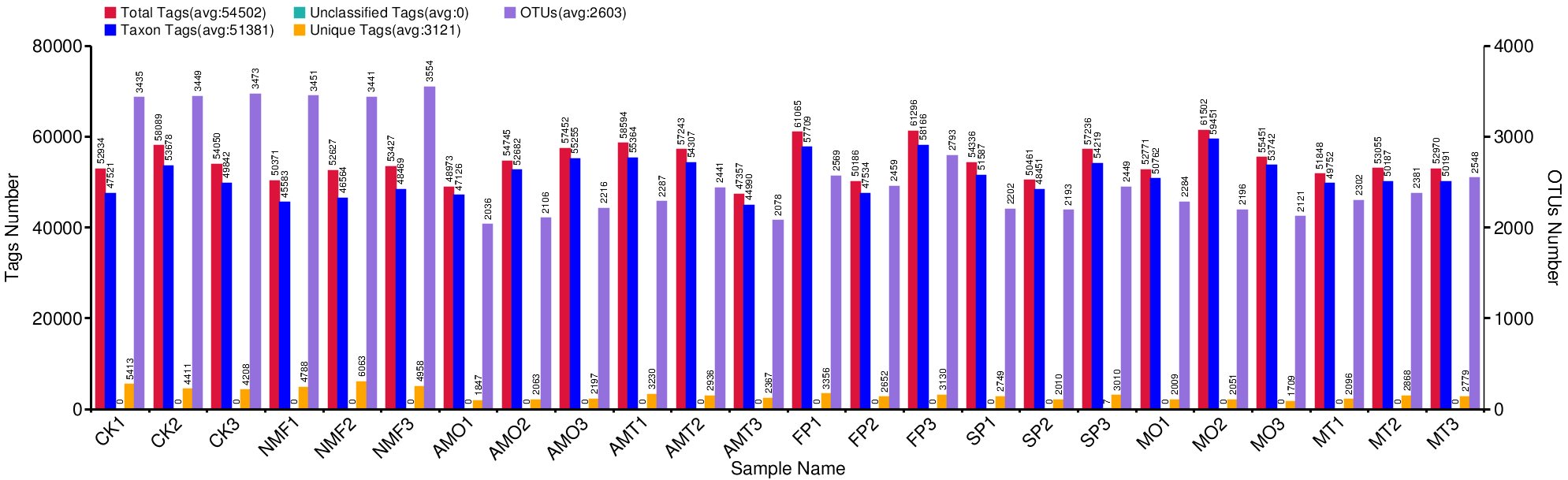


**Supplementary FIGURE S3 | Statistics of OTU cluster and species annotation for individual samples.** Total Tags (red columns): represent the total number of effective tags; Unique Tags (orange columns): represent the total numbers of singletons, which were removed from the dataset before further analysis; Taxon Tags (blue columns): represent the total numbers of tags subjected to OTU cluster and with species annotation; Unclassified Tags (green columns): represent the total numbers of tags without species annotation; OTUs (purple columns): represent the OTUs numbers for each sample. CK, FP and SP represent the control with no *P. heterophylla* cultivation, the newly planted and two-year monocultured plots, respectively. AMO, AMT represent the one-year cultivated plots that treated with equal amounts of microbial fertilizers NO.1, NO.2 for one month, respectively. NMF represents the one-year cultivated plots without microbial fertilizer treatment and was sampled at the same time as AMO and AMT. MO and MT represent the plots treated with microbial fertilizers NO.1 and NO.2 for seven months, respectively.


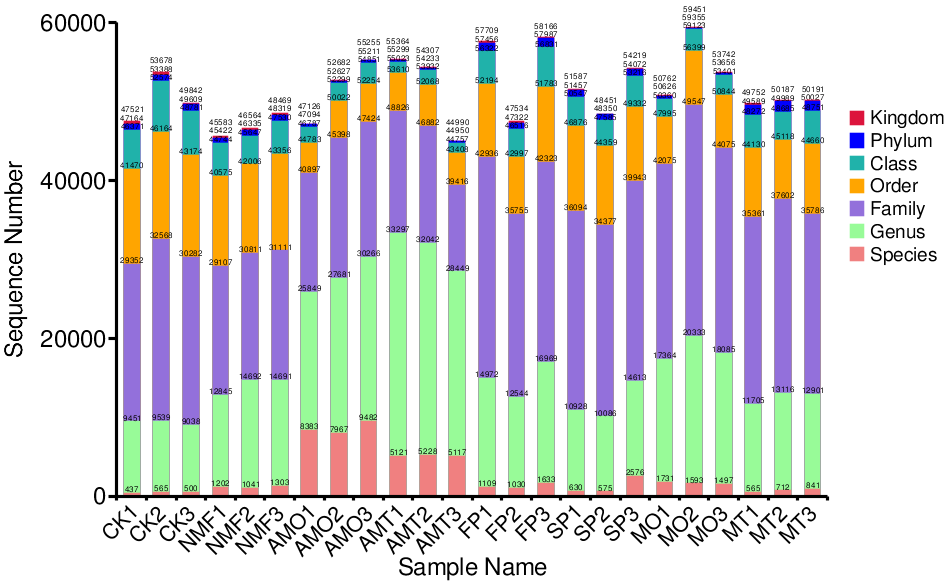


**Supplementary FIGURE S4 | Sequence numbers for each taxonomic level in 24 soil samples.** CK, FP and SP represent the control with no *P. heterophylla* cultivation, the newly planted and two-year monocultured plots, respectively. AMO, AMT represent the one-year cultivated plots that treated with equal amounts of microbial fertilizers NO.1, NO.2 for one month, respectively. NMF represents the one-year cultivated plots without microbial fertilizer treatment and was sampled at the same time as AMO and AMT. MO and MT represent the plots treated with microbial fertilizers NO.1 and NO.2 for seven months, respectively.


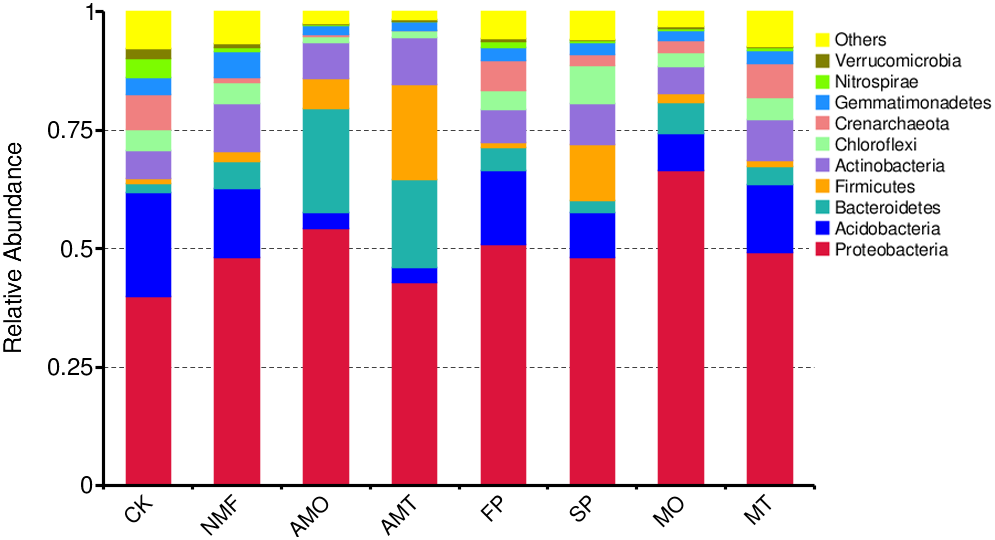


**Supplementary FIGURE S5 | Relative abundances of the top 10 baterial phyla in eight different soil samples.** CK, FP and SP represent the control with no *P. heterophylla* cultivation, the newly planted and two-year monocultured plots, respectively. AMO, AMT represent the one-year cultivated plots that treated with equal amounts of microbial fertilizers NO.1, NO.2 for one month, respectively. NMF represents the one-year cultivated plots without microbial fertilizer treatment and was sampled at the same time as AMO and AMT. MO and MT represent the plots treated with microbial fertilizers NO.1 and NO.2 for seven months, respectively.

**Supplementary TABLE S1 | Taxon-specific primer sets and their annealing temperatures for quantitative PCR.**

| **Target group** | **Primer** | **Sequence (5´- 3´)** | **Annealing temperature (°C)** | **Reference** |
| --- | --- | --- | --- | --- |
| *Fusarium oxysporum* | ITS1-F | CTTGGTCATTTAGAGGAAGTAA | 58 | Lievens *et al*., 2005 |
|  | AFP308R | CGAATTAACGCGAGTCCCAA |  | Lievens *et al*., 2005 |
| *Burkholderia* spp. | Burk3 | CTGCGAAAGCCGGAT | 55 | Drigo *et al*., 2009 |
|  | BurkR | TGCCATACTCTAGCYYGC |  | Drigo *et al*., 2009 |
| *prnD* gene | PRND1 | GGGGCGGGCCGTGGTGATGGA | 67 | Garbeva *et al*., 2004 |
|  | PRND2 | YCCCGCSGCCTGYCTGGTCTG |  | Garbeva *et al*., 2004 |
| Fungi | ITS1F | CTTGGTCATTTAGAGGAAGTAA | 59.5 | Manter *et al*., 2007 |
|  | ITS4 | TCCTCCGCTTATTGATATGC |  | Manter *et al*., 2007 |
| Bacteria | Eub 338 | ACTCCTACGGGAGGCAGCAG | 53 | Trivedi *et al*., 2012 |
|  | Eub 518 | ATTACCGCGGCTGCTGG |  | Trivedi *et al*., 2012 |

**REFERENCES**

Drigo, B., Van Veen, J.A., and Kowalchuk, G.A. (2009). Specific rhizosphere bacterial and fungal groups respond differently to elevated atmospheric CO_2_. *ISME J.* 3, 1204-1217. doi: 10.1038/ismej.2009.65

Garbeva, P., Voesenek, K., and van Elsas, J.D. (2004). Quantitative detection and diversity of the pyrrolnitrin biosynthetic locus in soil under different treatments. *Soil Biol. Biochem.* 36, 1453-1463. doi: 10.1016/j.soilbio.2004.03.009

Lievens, B., Brouwer, M., Vanachter, A.C.R.C., Levesque, C.A., Cammue, B.P.A., and Thomma, B.P.H.J. (2005). Quantitative assessment of phytopathogenic fungi in various substrates using a DNA macroarray. *Environ. Microbiol.* 7, 1698-1710. doi: 10.1111/j.1462-2920.2005.00816.x

Manter, D.K., and Vivanco, J.M. (2007). Use of the ITS primers, ITS1F and ITS4, to characterize fungal abundance and diversity in mixed-template samples by qPCR and length heterogeneity analysis. *J. Microbiol. Meth.* 71, 7-14. doi: 10.1016/j.mimet.2007.06.016

Trivedi, P., He, Z.L., Van Nostrand, J.D., Albrigo, G., Zhou, J.Z., aWang, N. (2012). Huanglongbing alters the structure and functional diversity of microbial communities associated with citrus rhizosphere. *ISME J.* 6, 363-383. doi: 10.1038/ismej.2011.100
